# Cytokine regulation of apoptosis-induced apoptosis and apoptosis-induced cell proliferation in vascular smooth muscle cells

**DOI:** 10.1007/s10495-020-01622-4

**Published:** 2020-07-05

**Authors:** Dimitra Aravani, Kirsty Foote, Nichola Figg, Alison Finigan, Anna Uryga, Murray Clarke, Martin Bennett

**Affiliations:** Division of Cardiovascular Medicine, University of Cambridge, ACCI, Addenbrooke’s Hospital, Box 110, CB2 0QQ Cambridge, UK

**Keywords:** Apoptosis, Proliferation, Vascular smooth muscle, Cytokine

## Abstract

Vascular smooth muscle cells (VSMCs) are the main structural cell of blood vessels, and VSMC apoptosis occurs in vascular disease, after injury, and in vessel remodeling during development. Although VSMC apoptosis is viewed as silent, recent studies show that apoptotic cells can promote apoptosis-induced compensatory proliferation (AICP), apoptosis-induced apoptosis (AIA), and migration of both local somatic and infiltrating inflammatory cells. However, the effects of VSMC apoptosis on adjacent VSMCs, and their underlying signaling and mechanisms are unknown. We examined the consequences of VSMC apoptosis after activating extrinsic and intrinsic death pathways. VSMCs undergoing apoptosis through Fas/CD95 or the protein kinase inhibitor staurosporine transcriptionally activated interleukin 6 (IL-6) and granulocyte-macrophage colony stimulating factor (GM-CSF), leading to their secretion. Apoptosis induced activation of p38MAPK, JNK, and Akt, but neither p38 and JNK activation nor IL-6 or GM-CSF induction required caspase cleavage. IL-6 induction depended upon p38 activity, while Fas-induced GM-CSF expression required p38 and JNK. Conditioned media from apoptotic VSMCs induced VSMC apoptosis in vitro, and IL-6 and GM-CSF acted as pro-survival factors for AIA. VSMC apoptosis was studied in vivo using SM22α-DTR mice that express the diphtheria toxin receptor in VSMCs only. DT administration induced VSMC apoptosis and VSMC proliferation, and also signficantly induced IL-6 and GM-CSF. We conclude that VSMC apoptosis activates multiple caspase-independent intracellular signaling cascades, leading to release of soluble cytokines involved in regulation of both cell proliferation and apoptosis. VSMC AICP may ameliorate while AIA may amplify the effects of pro-apoptotic stimuli in vessel remodeling and disease.

**Electronic supplementary material:**

The online version of this article (10.1007/s10495-020-01622-4) contains supplementary material, which is available to authorized users.

## Introduction

Vascular smooth muscle cells (VSMCs) are the main structural cell of blood vessels, and damage or death of VSMCs contributes to multiple vascular pathologies. In particular, apoptosis of VSMCs has been described in atherosclerosis (which underlies most heart attacks and strokes), arterial aneurysm, after injury (e.g. percutaneous coronary intervention) and in development. Although chronic apoptosis can reduce VSMC number [1], in many cases VSMC apoptosis is accompanied by changes in cell proliferation, cell migration, inflammation or further apoptosis, for example in developmental closure of the ductus arteriosus [2], vessel remodeling after changes in blood flow [3–5], vein grafting to arteries [6], and after injury [7]. However, in most cases the intracellular pathways triggered by either extrinsic (e.g. death receptor) or intrinsic apoptosis pathways, or their downstream consequences are not known.

VSMC proliferation after injury has been ascribed to exposure to mitogens released from the circulation (e.g. Platelet-derived growth factor from platelets) or vessel wall (e.g. Fibroblast growth factor). In contrast, VSMCs in development or after birth appear to proliferate and remodel the artery after apoptosis without exposure to these injury-induced cytokines. Similarly, VSMC apoptosis after injury is extensive, and has been ascribed to the injury stimulus; however, it’s not clear if the dead cells signal to induce apoptosis of adjacent cells. Although the conventional view is that VSMC apoptosis is silent, recent studies have suggested that apoptotic cells secrete cytokines or membrane-bound ligands; such paracrine effects can promote proliferation (apoptosis-induced (compensatory) proliferation, AICP)[8], apoptosis-induced apoptosis (AIA)[9], or inflammation, ameliorating or amplifying the effects of small numbers of dead cells in tissues. Furthermore, fundamental parts of the apoptosis machinery including caspase enzymes have both apoptotic and mitogenic roles simultaneously, coupling cell loss with proliferation of adjacent cells (reviewed in [10,11]). Remarkably, cells can also tolerate a certain threshold activation of apoptosis without compromising their viability, such that the same cell can be instructed to divide (autonomous proliferation), for example by caspase cleavage of an inhibitor of proliferation such as p21 [12].

We have examined the signaling pathways and consequences of VSMC apoptosis induced by both extrinsic and intrinsic apoptosis pathways in vitro, and VSMC apoptosis in vivo. VSMC apoptosis is associated with activation of a cascade of intracellular proteins and secretion of specific cytokines. These cytokines regulate VSMC apoptosis in culture, but VSMC apoptosis can also induce VSMC proliferation in vivo. VSMC AICP may ameliorate, while AIA may amplify the effects of pro-apoptotic stimuli in vessel remodeling and disease.

## Materials and methods

### Isolation and culture of mouse vascular smooth muscle cells (VSMCs)

VSMCs were isolated from mouse aorta by digesting with 1 mg/mL collagenase (Gibco, 17104-019) and 1 U/ml elastase (LS006365, Worthington) in serum-free Dulbecco’s Modified Eagle Medium (DMEM)(D5671, SIGMA), supplemented with 2 mM L-glutamine (G7513, SIGMA), 100U/mL penicillin and 100 µg streptomycin (P0781, SIGMA) for 10 min at 37 °C. Adventitia was removed and the remaining tissue digested in 2.5 mg/ml collagenase and 2.5 U/ml elastase for 2 h with regular trituration. Cells were further passaged and maintained in complete DMEM with 10% FCS at 37 °C and 5% CO_2_ in humidified conditions.

### Apoptosis assays

Cultured VSMCs were treated with either 1µM staurosporine (Stau)(Sigma, S4400) for 24 h in serum-free DMEM or 10 ng/ml α-Fas (BD Pharmingen 554255) + 10 nM cycloheximide (CHX)(Sigma, C7698) for 15 h in serum-free DMEM.

### Flow cytometry

Apoptosis was assessed by flow cytometry by Annexin-V (AV) and Propidium iodide (PI). Briefly 1 × 10^5^ cells were seeded in 6-well plates and treated with either Stau or α-Fas/CHX for 24 and 15 h respectively. Cells were trypsinized and stained for AV and PI using the FITC Annexin V/Dead Cell Apoptosis Kit (Thermo, V13242) according to the manufacturer’s instructions. A BD Accuri C6 flow cytometer was used to acquire the data and for data analysis. Gating was set according to live unstained VSMCs growing in serum-free DMEM (negative control for apoptosis).

### Fluorescence-activated cell sorting of apoptotic cells

72 × 10^5^ cells were seeded in T75 flasks and treated with 1µM Stau or 10 ng/ml α-Fas + 10 nM CHX for 24 and 15 h respectively. Cells were collected and stained for AV and PI using the FITC Annexin V/Dead Cell Apoptosis Kit (V13242, Thermo). AV^−/−^, AV^+^/PI^−^ and AV^+^/PI^+^ cell subpopulations were sorted, singlet discrimination applied, and collected in PBS for RNA extraction using a BD FACSAria III sorter. Gating was set according to the following controls; Stau-treated cells AV stained only (AV positive control), Stau-treated cells PI stained only (PI positive control), and live cells unstained (negative control).

### Western blotting

Attached apoptotic cells were collected and lysed in 50µ l RIPA buffer (Thermo) supplemented with 1X protease and phosphatase inhibitors (Merck, 539131, 524625) and denatured in 1X NuPAGE LDS containing 1X reducing agent (Thermo). 5 µg protein was resolved on pre-cast polyacrylamide 4–12% Bolt Bis-Tris Plus gels (Invitrogen) and transferred to a polyvinylidene difluoride membrane (PVDF) membrane by wet transfer using the Mini Trans-Blot Module (Biorad). Antibodies used for immunoblotting were from Cell Signaling Technology unless otherwise stated: p38 (9212S), P-p38 (9211S), JNK (9252), P-JNK (4668), Akt (4691S), P-Akt (4060S), cleaved caspase 3 (9661S), STAT3 (9139), P-STAT3 (9145), STAT5 (D206Y), P-STAT5 (Y694), MK2 (3042), P-MK2 (3041), c-Jun (9165), P-c-Jun (3270), β actin (Sigma, A5441-clone AC15), Anti-mouse IgG-HRP (7076), Anti-rabbit IgG- HRP (7074). Blots were visualized by chemilluminescence (GE Healthcare).

### Cell treatments

VSMCs were pre-treated for 1 h with 50µM Z-VAD.FMK (R&D Systems FMK001) prior to addition of Stau and α-Fas/CHX and incubated for the indicated time points. VSMCs were also pre-treated with 10µM SB203580 (CELL, SM32) and 25µM SP600125 (Sigma, S5567). Recombinant proteins were purchased from Peprotech. Cells were treated with 100 ng/ml IL-6 (216 − 16) and GM-CSF (315-03) or 50 ng/ml of each for combined treatment. Neutralization of IL-6 and GM-CSF in conditioned medium was achieved with 0.75 µg/ml of anti-GM-CSF (R&D MAB415-SP) and anti-IL-6 (R&D MAB406-SP). Inhibition of transcription was performed with 2 µg/ml of Actinomycin-D (NovusBio, NB1229).

### Preparation of conditioned medium

VSMCs were treated with Stau, α-Fas/CHX or DMSO (vehicle control) in serum-free DMEM for 8 h. Cells were then washed twice in PBS and incubated in serum-free DMEM for 15 h. Cell supernatants were cleared at 11,000x rpm for 5 min and concentrated using Vivaspin columns (Generon UK) with an exclusion range of 5 kDa. Concentrated conditioned medium was stored at − 80 °C until further use.

### Cell cycle analysis

1 × 10^5^ cells were seeded in 6-well plates in DMEM + 10% FBS and left to attach overnight. Following 48 h incubation in serum-free DMEM, cells were further treated for 24 h with concentrated conditioned medium from Stau, α-Fas/CHX or DMSO-treated donor cells supplemented with 10µM EdU. Cells were collected by trypsinization and stained for EdU, followed by RNase (100 µg/ml) and PI (20 µg/ml) treatment as per manufacturer’s instructions (Invitrogen, C10632). A BD Accuri C6 flow cytometer was used to acquire the data and for data analysis.

### Immunohistochemistry

Paraffin-embedded tissues were sectioned at 5 µm intervals. Specimens were de-waxed and rehydrated through graded ethanols to water and microwaved in 120 mM sodium citrate buffer. Endogenous peroxidase activity was blocked with 3% hydrogen peroxide. Sections were then incubated in 10% bovine serum albumin in PBS for 1 h at RT and then with primary antibodies including MAC3 (BD Biosciences, 553322, 1:500), Ki67 (Abcam, ab16667) overnight at 4 °C. HRP-conjugated secondary antibodies were applied on the second day and visualized using DAB. Incorporation of dUTPdigoxigenin (TUNEL assay for apoptosis) was detected with an alkaline phosphatase-conjugated antibody to digoxigenin (Roche) and development with 5-bromo-4-chloro-3indoyl-phosphate/p-nitroblue tetrazolium (Vector). Quantification was performed as a percentage of positively stained cells against total number of cells (3 sections used per mouse).

### ELISA

Cell culture supernatants were concentrated ~ 10 times on columns and subjected to bead-based immunoassays according to the manufacturer’s instructions (BD 558301, 558347, 558296, 558299 560232). Data Acquisition and analysis was performed using a BD Accuri C6 flow cytometer and the eBioscience FlowCytomixTM Pro software respectively.

### Multiplex cytokine array

Cell culture supernatants were concentrated ~ 10 times on columns and subjected to a magnetic bead-based multianalyte Luminex assay (R&D systems) according to the manufacturer’s instructions. Data acquisition was performed in a Luminex Magpix.

#### qPCR

RNA was extracted from mouse aorta and VSMCs and then reverse-transcribed into cDNA using the cDNA synthesis kit (BIOLINE, BIO-65053). Expression analysis was performed for IL-6 (forward primer 5′- CTCTGCAAGAGACTTCCATCCA − 3′ and reverse primer 5′- AGTCTCCTCTCCCGGACTTGT-3′), GM-CSF (forward 5′TGCCTGTCACGTTGAATGAAG-3′ and reverse 5′- GAAATTGCCCCGTAGACCCT-3′), TGFβ (forward 5′AACGGGATCAGCCCCAAAC-3′ and reverse 5′- TCTCTGTGGAGCTGAAGCAA-3′), IGFBP-3 (forward 5′-CACACCGAGTGACCGATTCC-3′ and reverse 5′- GTGTCTGTGCTTTGAGACTCAT-3′) and IL-18 (forward primer- 5′TCTTGGCCCAGGAACAATGG-3′ and reverse 5′- ACAGTGAAGTCGGCCAAAGT-3′primer sequences). Comparative quantitation was performed using the Rotor-Gene software. For each experimental sample the relative control sample was used as calibrator and all samples were normalized to either Rpl4 (forward 5′- CGCAACATCCCTGGTATTACT-3′ and reverse 5′-ACTTCCGGAAAGCACTCTCCG-3′) or 18S (forward 5′- GTAACCCGTTGAACCCCATT-3′ and reverse 5′-CCATCCAATCGGTAGTAGCG-3′).

### SM22α-hDTR mice

All animal studies were undertaken under UK Home Office licensing and approved by the Animal Welfare Ethical Review Body of the University of Cambridge. C57BL6/J SM22α-hDTR mice were generated and genotyped as previously described [13]. Male and female SM22α-hDTR mice and wild type littermates were treated as indicated with intraperitoneal doses of 5 ng/g body weight diphtheria toxin (LIST biological laboratories, 150) diluted in 0.9% saline and 0.2% BSA.

### Carotid ligation expriments

Mice (C57BL6/J SM22α-hDTR /male and female) were given a pre-operative injection of buprenorphine (0.1 mg/kg; subcutaneously) and anaesthetized with 2.5% inhalable isofluorane (maintained at 1.5%). The left common carotid artery was exposed and ligated just below the bifurcation with a 6 − 0 silk suture. Mice were permitted to recover for 14d and then sacrificed for collection of right and left common carotid arteries. Tissues were fixed in 10% neutral-buffered formalin overnight before paraffin embedding.

### Statistical analysis

Results are presented as mean ± SD unless stated otherwise. Two-sample unpaired Student t tests were used for variables that were normally distributed and compared between 2 groups. If the variable was not normally distributed, we conducted a Mann–Whitney test. All statistical analyses were performed with GraphPad Prism 7.00 (GraphPad Software Inc).

## Results

### Activation of extrinsic or intrinsic apoptosis of VSMCs

**Fig. 1:**
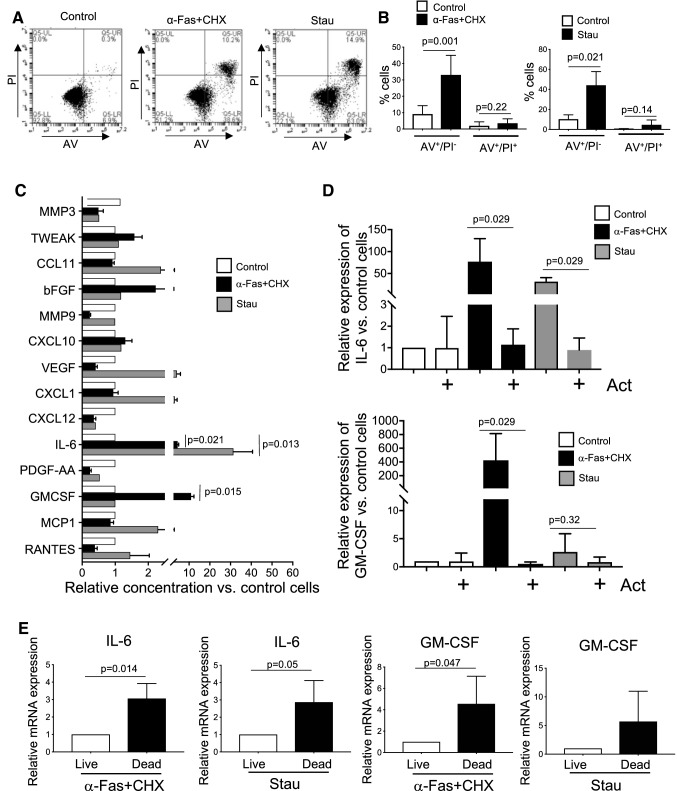
Fas activation and staurosporine induce apoptosis and secretion of soluble cytokines **a** Flow cytometric profiles of mouse VSMCs treated with α-Fas + 10 nM cycloheximide (CHX) or 1µM staurosporine (Stau) for 15 and 24 h respectively stained for annexin V (AV) or propidium iodide (PI). **b** % AV^+^/PI^−^ or AV^+^/PI^+^ cells after treatment in **a**. **c** Cytokine array for secreted cytokines from cells treated in **a**. **d** Relative mRNA expression of IL-6 or GM-CSF mRNA in cells pre-treated with Actinomycin D (ActD) and then treated with α-Fas + CHX or Stau, n = 4. **e** Relative mRNA expression in live (AV^−^/PI^−^) or dying/dead (AV^+^/PI^−^ or AV^+^/PI^+^) sorted cells treated as above Data are means ± SD, n = 3 unless otherwise indicated

To identify pathways and cytokines released when VSMCs undergo apoptosis, we treated mouse VSMCs with an agonistic antibody to the death receptor CD95/Fas and 10 nM cycloheximide (α-FAS + CHX), a well-characterized activator of extrinsic apoptosis, or the protein kinase inhibitor staurosporine (Stau), a similarly well-established activator of intrinsic apoptosis, both in 0% serum media. 10 nM cycloheximide is well below concentrations that result in global protein synthesis inhibition (~ 35µM)[14]. Apoptosis was quantified by flow cytometry using propidium iodide (PI) and FITC-annexin V (AV). Control VSMCs in 0% FCS showed low levels of both apoptosis (AV^+^PI^−^) and secondary necrosis (AV^+^PI^+^), while both α-FAS + CHX and staurosporine rapidly induced cell death with apoptosis (AV^+^/PI^−^) predominating at 15 and 24 h respectively (Fig. 1a-b).

### VSMC apoptosis induces synthesis and release of IL-6 and GM-CSF

To determine the soluble cytokines released during VSMC apoptosis, we examined conditioned media of apoptotic cells and the adjacent live cells by multiplex cytokine array, and quantified relative concentrations of specific cytokines by ELISA. The major cytokine released following both α-Fas + CHX and staurosporine was IL-6, while GM-CSF was also released by α-Fas + CHX (Fig. 1c). To determine whether synthesis of these cytokines was increased by apoptosis, or apoptosis just caused release from pre-formed stores, VSMCs were incubated with actinomycin D (ActD) prior to induction of apoptosis, and IL-6 and GM-CSF mRNA assayed by qPCR. α-Fas + CHX and staurosporine markedly increased IL-6 mRNA, while α-Fas + CHX increased GM-CSF compared with control-treated cells. Expression of these cytokine mRNAs was completely inhibited by ActD (Fig. 1d), indicating that α-Fas + CHX and staurosporine increase IL-6 and GM-CSF in conditioned media in part by transcriptional activation.

The cell cultures used above contain cells undergoing apoptosis, secondary necrosis, and adjacent live cells, and it is therefore unclear whether IL-6 and GM-CSF are released from the dying (apoptotic or secondary necrotic) or live cells, or both. For example, both IL-6 and GM-CSF are released from VSMCs when stimulated with pro-inflammatory cytokines such as IL-1α [15]. We therefore sorted VSMCs after α-Fas + CHX or staurosporine treatment using annexin V/PI into dying or dead cells (AV^+^/PI^−^ and AV^+^/PI^+^) or live cells (AV^−^/PI^−^), and examined cytokine expression by qPCR. IL-6 and GM-CSF mRNA expression were increased in the population undergoing cell death compared with similarly-treated live cells (Fig. 1e).

### Apoptosis induces phosphorylation of p38, JNK, and Akt

Fas activation induces receptor aggregation, clustering of adapter molecules such as FADD (Fas-associated death domain), and activation of a number of downstream signaling pathways leading to apoptosis, including activation and cleavage of caspases 3, 6, 7 and 8. However, Fas activation also leads to activation of kinase cascades involving mitogen-activated protein kinase kinase kinase 1 (MEKK1), Jun amino (N)-terminal kinase kinase 1 (JNKK1), p38 mitogen-activated protein kinase (p38MAPK) and Akt/protein kinase B (PKB)[16]. The downstream targets of these cascades include Nuclear Factor kappa-light-chain-enhancer of activated B cells (NF-κF) and Activator protein 1 (AP1 sites) on many promoters, including a number of VSMC mitogens and apoptosis regulatory proteins (reviewed in [16, 17]). Similarly, staurosporine induces apoptosis via effects on a wide range of pro-survival and pro-apoptotic protein kinases [18], some of which may also promote proliferation or apoptosis. Thus, pathways leading to apoptosis and proliferation may be stimulated simultaneously in the same cell or sequentially in adjacent cells by both agents. However, it is unclear whether synthesis and release of soluble cytokines requires caspase cleavage, or is just a by-product of simultaneous activation of common upstream pathways.

**Fig. 2:**
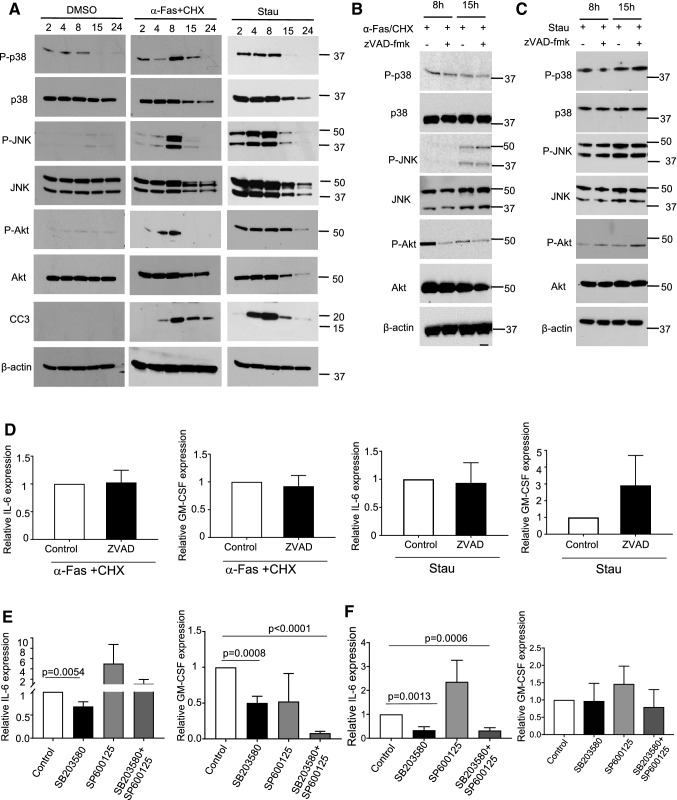
Apoptosis induces activation of p38, JNK and Akt. **a** Western blots of mouse VSMCs treated with DMSO (control diluent), α-Fas + CHX or Staurosporine for 2–24 h for p38, JNK, Akt, cleaved caspase 3 (CC3), or β-actin. **b**, **c** Western blot of VSMCs treated with α-Fas + CHX. **b** or Stau **c** ± pre-incubation with 50µM ZVAD-fmk. **d** qPCR for IL-6 or GM-CSF of cells treated in **b**, **c**. n = 4. **e, f** qPCR for IL-6 or GM-CSF of cells pre-treated with inhibitors to p38 (SB203580, 10µM) or JNK (SP600125, 25µM) alone or in combination and apoptosis induced by α-Fas + CHX **e** or Staurosporine **f**. Data are means ± SD, n = 3

We therefore examined the time course of phosphorylation of p38MAPK, JNK and Akt compared with their unphosphorylated forms, and cleavage of caspase 3 after treatment with α-Fas + CHX or staurosporine. p38 showed some phosphorylation in DMSO-treated cells, but P-p38 increased significantly after α-Fas + CHX or staurosporine, peaking at 8 h. P-JNK also increased after both apoptotic stimuli, peaking at 8 h, while P-Akt was increased by staurosporine but more variably and not significantly by α-Fas + CHX (Fig. 2a)(Supplemental Fig. 1). Cleaved Caspase 3 (CC3) was seen with both α-Fas + CHX and staurosporine, peaking at 8 h and then reduced as cell death proceeded (Fig. 2a).

To determine whether caspase cleavage was required for activation of p38, JNK or Akt, we incubated cells induced to undergo apoptosis with the broad spectrum caspase inhibitor ZVAD.fmk and studied P-p38, P-JNK and P-Akt at 8 and 15 h. Interestingly, ZVAD.fmk had no effect on α-Fas + CHX or staurosporine-induced p38 or JNK activation and a variable effect on P-Akt (Fig. 2b-c). Furthermore, ZVAD.fmk did not reduce α-Fas + CHX or staurosporine-induced induction of IL-6 and GM-CSF mRNA expression (Fig. 2d), suggesting that Fas and staurosporine-induced transcription of IL-6 and GM-CSF does not require caspase activation.

### IL-6 induction requires p38 activity

We next examined whether synthesis of IL-6 and GM-CSF during apoptosis requires activation of p38 or JNK, using the p38MAPK inhibitor SB203580 or the JNK inhibitor SP600125, or both. SB203580 reduced staurosporine-induced phosphorylation of the p38MAPK target MK2 while SP600125 reduced staurosporine-induced phosphorylation of c-Jun (with also some reduction of total c-JUN, most likely from autoactivation)(Supplemental Fig. 2), confirming the inhibitory effect of these agents. Neither SB203580 nor SP600125 inhibited cleaved caspase 3 induced by staurosporine (Supplemental Fig. 2). SB203580 but not SP600125 inhibited both α-Fas + CHX and staurosporine-induced IL-6 mRNA expression, and SB203580 reduced α-Fas + CHX-induced GM-CSF mRNA expression (Fig. 2e-f).

These data show divergence of pathways activated by Fas and staurosporine, with different pathways leading to caspase cleavage to those leading to p38, and JNK activation, and induction of IL-6 and GM-CSF. In particular, IL-6 and GM-CSF induction are dependent upon p38 but not JNK activity, while caspase cleavage is not dependent upon either p38 or JNK.

### VSMC apoptosis induces apoptosis but not cell proliferation in adjacent VSMCs in vitro

IL-6 is a mitogen for VSMCs [19, 20], but can also regulate apoptosis [21], while GM-CSF has multiple activities on VSMCs, including induction of TGFβ which inhibits VSMC proliferation, and affects extracellular matrix (ECM) synthesis [22]. Mice deficient in GM-CSF show reduced arterial neointima formation after injury, in part by reduced macrophage accumulation and proliferation [23], and VSMCs and endothelial cells (ECs) are the major cell expressing GM-CSF in early atherogenesis [24, 22]. It is therefore unclear whether VSMC apoptosis induces proliferation, apoptosis, or neither in adjacent live VSMCs, and whether secreted IL-6 or GM-CSF are responsible.

**Fig. 3:**
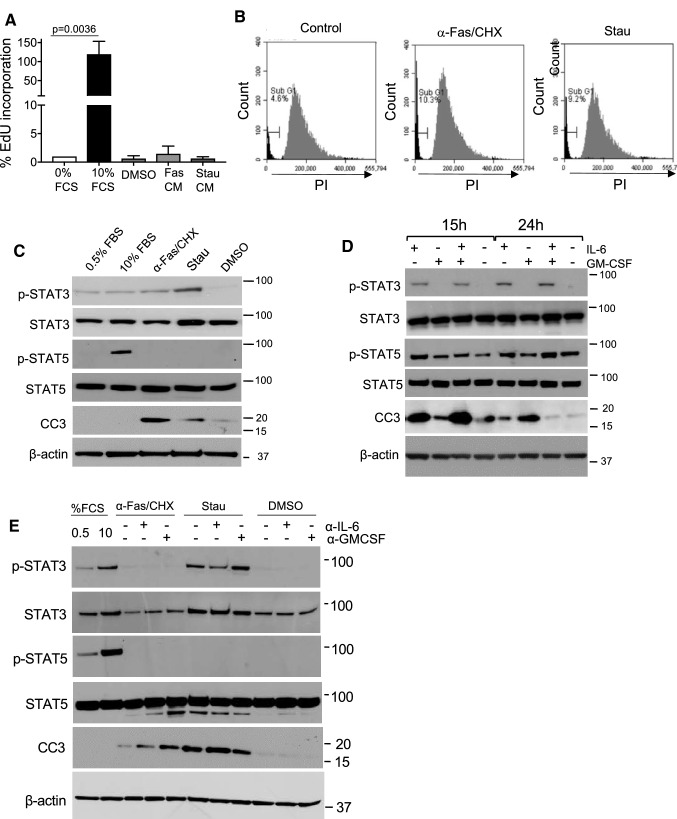
Apoptosis of VSMCs induces apoptosis in recipient VSMCs .**a** EdU incorporation of growth-arrested VSMCs after treatment with media containing 10% FBS, conditioned media from VSMCs induced to undergo apoptosis by α-Fas + CHX or Stau, or DMSO control. **b** Flow cytometric profiles of recipient cells after treatment with conditioned media from control donor cells or apoptotic VSMCs. **c** Western blot for STAT3, STAT5 and CC3 for recipient cells treated as in **a**. **d** Western blot for STAT3, STAT5 and CC3 for VSMCs treated with 100 ng/ml IL-6 or GM-CSF for 15 or 24 h alone or in combination (50 ng/ml each). **e** Western blot for STAT3, STAT5 and CC3 of recipient cells treated with conditioned media from apoptotic VSMCs ± 0.75µ g/ml neutralizing antibodies to IL-6 or GM-CSF. N = 3

We cultured recipient VSMCs in 0.5% FBS media for 72 h to induce quiescence. Apoptosis was induced in donor VSMCs with α-Fas + CHX or staurosporine, cells washed thoroughly after 8 h to remove α-Fas + CHX or staurosporine, fresh 0.5% FBS media replaced, and conditioned media collected. Subsequent culture of recipient VSMCs in 10% serum showed that they retained the ability to rapidly enter the cell cycle as determined by EdU incorporation. However, the conditioned media from either α-Fas + CHX or staurosporine-treated cells failed to induce cell cycle entry, even when concentrated 10-fold (Fig. 3a-b**)**. We also examined whether α-Fas + CHX or staurosporine-conditioned media could promote proliferation with a submaximal proliferative stimulus of 5% FBS. 5% FBS induced 1% EdU incorporation, but neither α-Fas + CHX nor staurosporine-conditioned media with 5% FBS increased % EdU incorporation above controls (Supplemental Fig. 3). This data suggests that the combination of soluble mitogens released after Fas or staurosporine-induced apoptosis in culture is not sufficient to induce cell proliferation alone, or augment proliferation with a submaximal proliferative stimulus of 5% FBS.

In contrast, flow cytometric profiles of recipient cells showed evidence of a sub-diploid peak that was increased by α-Fas + CHX or staurosporine-conditioned media from donor cells (Fig. 3b), suggestive of VSMC apoptosis-induced apoptosis (AIA). IL-6 and GM-CSF can have either pro- or anti-apoptotic actions, depending upon concentration, cell type and stimulus [25–29]. We therefore examined whether recipient cells showed evidence of intracellular signals induced by IL-6 or GM-CSF that regulate proliferation or apoptosis. STAT3 is a major signaling pathway that regulates the anti-apoptotic activity of IL-6 [25, 26], and STAT5 is also implicated in IL-6 signaling in VSMCs [30]. Similarly, STAT 3 and 5 regulate GM-CSF signaling in multiple cell types [31]. Recipient cells showed activation of STAT3 induced by conditioned media from donor cells treated with α-Fas + CHX or staurosporine. However, despite STAT3 phosphorylation, recipient cells showed increased caspase 3 cleavage when incubated in α-Fas + CHX or staurosporine-conditioned media. In contrast, α-Fas + CHX or staurosporine-conditioned media did not activate STAT5 in recipient cells, a proliferation signal seen after 10% FBS treatment (Fig. 3c).

### IL-6 and GM-CSF in conditioned media are pro-apoptotic

This data suggests that α-Fas + CHX or staurosporine-conditioned media contains a mixture of cytokines that possess predominantly pro-apoptotic activity, but are not sufficient to induce proliferation from quiescence. Although multiple cytokines were released after α-Fas + CHX or staurosporine treatment (Fig. 1), we first examined the effects of IL-6 and GM-CSF using either recombinant cytokines, or neutralizing antibodies on conditioned media-induced apoptosis. Recombinant IL-6 induced STAT3 but not STAT 5 phosphorylation and both IL-6 and GM-CSF could induce apoptosis, as indicated by cleavage of caspase 3, with a peak at 15 h (IL-6) or 24 h (GM-CSF) (Fig. 3d). Interestingly, IL-6 and GM-CSF together potently induced CC3 at 15 h, but the CC3 signal was lost by 24 h, most likely due to further CC3 degradation (Fig. 3d).

Although high concentrations of recombinant IL-6 or GM-CSF could induce VSMC apoptosis, we also tested the effects of neutralizing IL-6 and GM-CSF in the conditioned media. 2.5µ g/ml of a neutralizing antibody to IL-6 or GM-CSF effectively inhibited activation of STAT3 or STAT5 respectively as markers of IL-6 and GM-CSF signaling (Supplemental Fig. 4). However, the pro-apoptotic effect of the conditioned media from α-Fas + CHX or staurosporine-treated cells could not be inhibited by inhibiting IL-6 or GM-CSF. Indeed, neutralizing IL-6 or GM-CSF increased CC3 cleavage when cells were treated with conditioned media from α-Fas + CHX, and neutralizing IL-6 increased CC3 cleavage when cells were treated with conditioned media from staurosporine-treated VSMCs (Fig. 3e), suggesting that the levels of IL-6 and GM-CSF in conditioned media are anti-apoptotic.

This data suggests that the overall pro-apoptotic effect of α-Fas + CHX- or staurosporine-conditioned media is likely to be due to relative changes in a large number of secreted pro-and anti-apoptotic cytokines. Indeed, combinations of pro-inflammatory cytokines including IL-1α, TNFα and Interferon-gamma (IFN-γ can induce VSMC apoptosis [32, 33] in part by Fas signaling [34]. However, IL-1α, TNFα and IFNγ were not detectable in α-Fas + CHX or staurosporine-conditioned media. In addition, there were no consistent changes in the pro-apoptotic cytokines IL-18 or insulin-like growth factor binding protein 3 (IGFBP3), or the anti-apoptotic cytokine TGFβ (Supplemental Fig. 5).

### VSMC apoptosis induces IL-6 and GM-CSF and cell proliferation in normal arteries in vivo

**Fig. 4:**
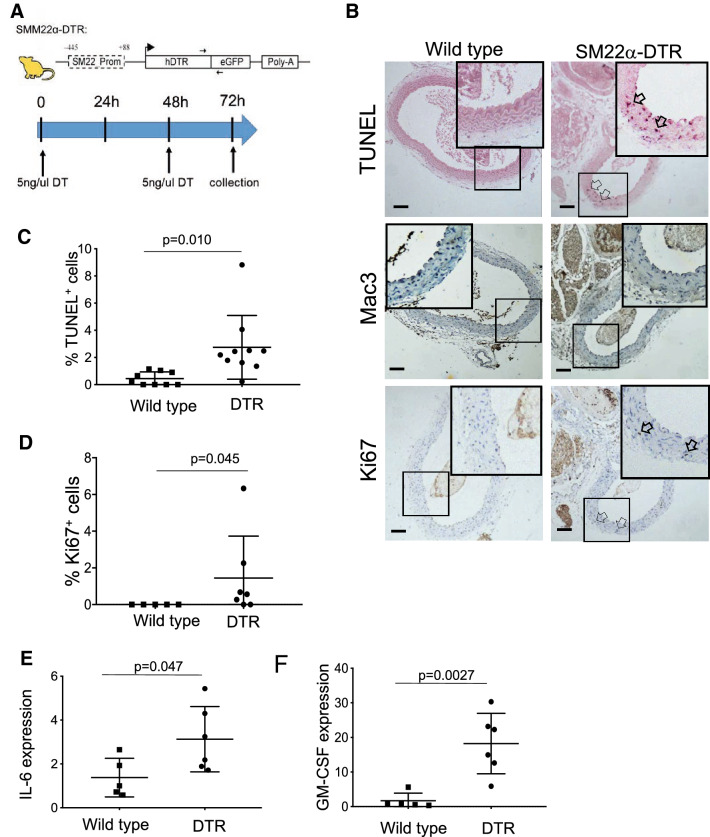
VSMC apoptosis induces VSMC proliferation in vivo. **a** Schematic of SM22α-DTR transgenic mice and DT administration protocol. DT was administered to SM22α-DTR (DTR) or wild-type littermate control (wild type) mice for 48 h and aortas collected at 72 h. **b** TUNEL, or immunohistochemistry for Mac3 or Ki67 in wild-type control or SM22α-DTR mice treated with DT for 48 h and sections collected at 72 h. Insets show high power views of areas outlined. Scale bar = 100µ m. **c-d** % TUNEL^+^ **c** or Ki67^+^ **d** VSMCs in media of mice in **b**. n = 7–9. **e-f** qPCR for IL-6 or GM-CSF mRNA in media of vessels in **b** relative to Rpl4. n = 5–6. Data are means ± SD. Scale bars = 100µ m

Although conditioned media from apoptotic VSMCs induced apoptosis rather than cell cycle entry of live VSMCs in culture, the effect of VSMC apoptosis on adjacent VSMCs in vivo is unclear. For example, VSMCs in vivo are in a stable growth-arrested state, unlike cultured VSMCs that have undergone transition to a phenotype that shows increased proliferation and susceptibility to apoptosis. In addition, VSMC proliferation and apoptosis in vivo may be triggered by membrane-bound proteins [35] or proteins contained within exosomes [36], which are not present in filtered conditioned media. We therefore examined transcriptional induction of cytokines and proliferation of VSMCs in the vessel wall following induced VSMC apoptosis. SM22α-DTR mice express the human diphtheria toxin receptor (DTR) from the minimal SM22α promoter (Fig. 4a). Mice are normally resistant to DT, but transgenic expression of DTR renders cells susceptible to DT-induced apoptosis. DT administration to SM22α-DTR mice results in apoptosis of medial or intimal VSMCs in normal vessels, after injury, or in atherogenesis [1, 5, 13, 37]. Furthermore, DT administration to VSMCs derived from SM22α-DTR mice causes release of IL-6, MCP-1 and GM-CSF in culture, and in the presence of hyperlipidemia, increases serum levels of TNF-α and IL-6 in vivo [37].

We administered DT to SM22α-DTR or wild-type littermate control mice for 48 h. Aortas were collected 24 h later and apoptosis examined by TUNEL, IL-6 and GM-CSF mRNA by qPCR, and cell proliferation by immunohistochemistry for Ki67. DT induced apoptosis in SM22α-DTR but not wild type control mice, with no infiltration of Mac3^+^ inflammatory cells (Fig. 4b-c). Cell proliferation was not detectable in wild type mice after DT administration, but small numbers of Ki67^+^ cells were found 24 h after DT administration in the media of SM22α-DTR mice (Fig. 4b,d). DT also induced IL-6 mRNA approximately 3-fold and GM-CSF approximately 20-fold in SM22α-DTR compared with wild type control mice (Fig. 4e-f).

### Induced VSMC apoptosis augments cell proliferation after vessel injury

**Fig. 5:**
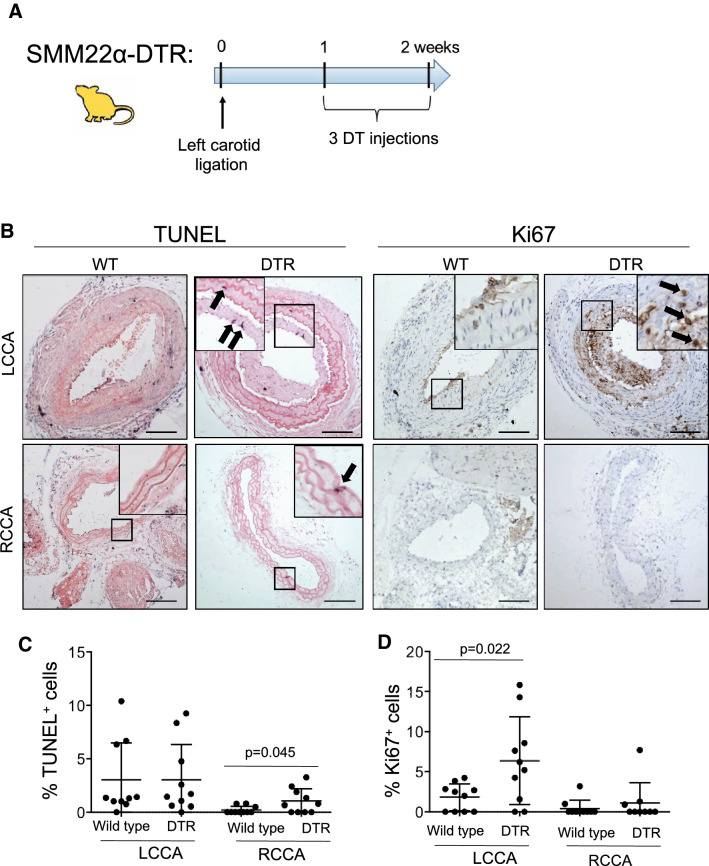
VSMC apoptosis induced after vessel injury augments cell proliferation. **a** Schematic of SM22α-DTR transgenic mice and DT administration protocol. The left common carotid artery was ligated and DT administered to SM22α-DTR (DTR) or wild-type littermate control mice (wild type) on 3 occasions from 1-2w. Carotid arteries were collected at 2w. **b-d** TUNEL or immunohistochemistry for Ki67 **b** or quantification **c-d** in left common carotid artery (LCCA) or right common carotid artery (RCCA) wild-type control or SM22α-DTR mice treated with DT. Insets show high power views of areas outlined. Scale bar = 100µ m. Data are means ± SD, n = 10

Vessel injury is associated with massive VSMC apoptosis, followed by repopulation of the media to recover its cellularity and intima formation through VSMC proliferation and migration. Although VSMC apoptosis can induce VSMC proliferation in normal arteries in vivo as above, it is unclear whether this local and relatively small effect can augment the proliferative actions of mitogens and release from extracellular matrix restraint that drives medial repopulation and intima formation after injury. We therefore examined the effect of DT-induced apoptosis after left common carotid artery (LCCA) ligation, a model of reduced flow-induced neointima formation, and in the right common carotid artery (RCCA), which undergoes compensatory high flow-induced remodeling, both of which are accompanied by apoptosis (Fig. 5a). The LCCA was ligated, mice administered DT 3X/week 1w later to both groups, and sections analysed at 2w. SM22α-DTR mice had increased apoptosis in the RCCA, but not in the ligated LCCA at 2w (Fig. 5b,c). In contrast, SM22α-DTR mice had increased proliferation in the LCCA but this was not seen in the RCCA (Fig. 5b,d).

## Discussion

While VSMC apoptosis occurs in normal development and a large range of vascular diseases, its effects have been presumed to be due to loss of VSMC function, in particular the structural integrity provided by synthesis or extracellular matrix proteins. In contrast, the direct effects of VSMC apoptosis, for example release of soluble mediators that can have large effects on adjacent live cells, has received less attention.

**Fig. 6:**
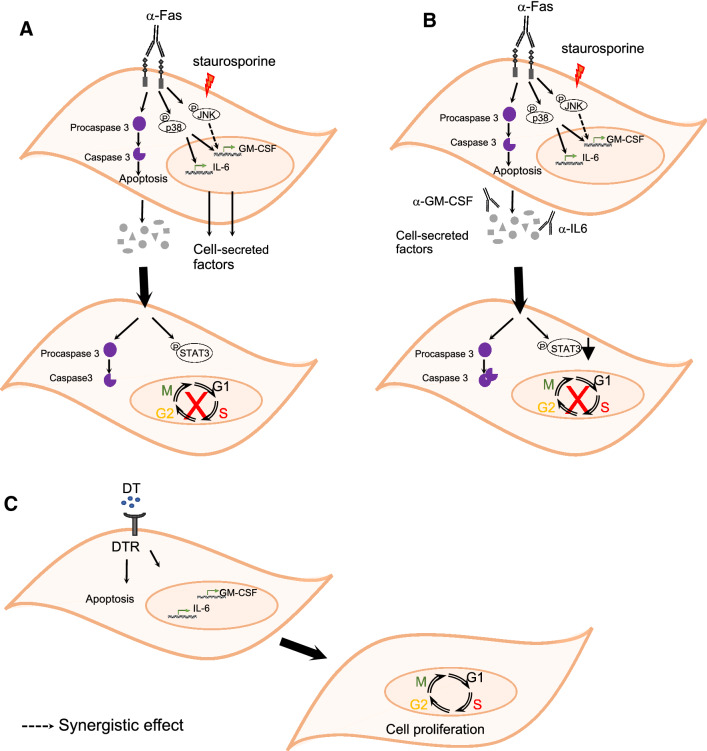
Schematic representation of mechanisms in VSMC apoptosis-induced apoptosis and apoptosis induced compensatory proliferation. **a** In vitro stimulation with either α-FAS/CHX or staurosporine induces apoptosis via cleavage of caspase 3 and transcription and secretion of IL-6 and GM-CSF (α-FAS/CHX) and GM-CSF (α-FAS/CHX or staurosporine). Conditioned media of apoptotic VSMCs induce cleavage of caspase 3 in recipient cells and phosphorylation of STAT3, but growth-arrested recipient cells do not enter the cell cycle. **b** Neutralization of IL-6 or GM-CSF in the conditioned medium causes cleavage of caspase 3 (α-IL-6 and α-GM-CSF) and reduction in p-STAT3 in recipient cells (α-IL6). **c** In vivo, DT-induces VSMC apoptosis in the vessel wall and transcriptional upregulation of IL-6 or GM-CSF, and proliferation of adjacent VSMCs

There are a number of novel and important findings in our study, summarised in Fig. 6. In particular we show that: (a) both extrinsic and intrinsic apoptosis signals induce mRNA of both IL-6 and GM-CSF in the dying VSMCs, which are then secreted: (b) VSMCs induced to undergo apoptosis activate multiple intracellular signaling cascades, including phosphorylation of p38, JNK and Akt, as well as activation of caspases: (c) caspases are not required for phosphorylation of p38, JNK and Akt, nor induction of IL-6 and GM-CSF: (d) p38 but not JNK activation is required for IL-6 and GM-CSF induction: (e) conditioned media from apoptotic VSMCs does not induce or augment proliferation of adjacent live VSMCs from quiescence in culture, but rather induces further apoptosis: (f) high concentrations of IL-6 and GM-CSF can induce VSMC apoptosis, but the concentrations in conditioned media from apoptotc VSMCs promote VSMC survival, and: (g) VSMC apoptosis induces IL-6 and GM-CSF in normal vessels in vivo, and increases VSMC proliferation both in normal vessels and after ligation. Apoptotic VSMCs are rapidly cleared in normal vessels and TUNEL only marks a portion of the apoptotic process; a TUNEL^+^ % of 2–3% therefore represents significant apoptosis. Similarly, VSMCs in normal vessels are in a quiescent (non-proliferating) state; detection of any proliferating VSMCs in otherwise normal vessels therefore represents a significant pro-proliferative stimulus.

Apoptosis-induced (compensatory) proliferation (AICP) is seen in normal development and tissue homeostasis in a wide variety of organisms and tissues (reviewed in [38, 39]). Although the precise regulation of AICP is not known, in almost all cases developmental AICP requires caspase activation, and some of the signaling pathways are conserved across species. For example, in *Drosophila* the initiator caspase 9 homologue Dronc can activate *Drosophila* p53 (Dp53), resulting in secretion of the mitogens Wingless (Wg) and Decapentaplegic (Dpp)[38, 39]. JNK signaling is implicated in both apoptosis and Wg and Dpp release, induced by a variety of stimuli [40].

Although these pathways are established in *Drosophila*, their role in mammalian cell AICP is unclear. Caspases are required for cytokine secretion in some systems [41][42] and bone morphogenetic protein-2 (BMP2) and Wnts, the human homologues of DPP2 and Wg respectively, can regulate VSMC proliferation and migration, although not always positively [43–45]. In contrast, Fas/FADD activation can induce a number of pro-inflammatory genes including MCP-1, IL-8, tumor-necrosis-factor-stimulated protein (TSG) -6, PAI 2, IL-6, GRO1 and IL-1α [46] while caspase inhibition could only partially, block upregulation of MCP-1 transcript expression, despite complete inhibition of apoptosis [46].

Mammalian cells also possess other AICP pathways not defined in other organisms. For example, caspases 3 and 7 cleave and activate the Ca^2+^- independent phospholipase A2 (iPLA2), resulting in release of prostaglandin E2 (PGE2)[41]. PGE2 promotes AICP in skin and liver [41], and can stimulate proliferation of quiescent VSMCs [47]. PGE2 can also activate Wnt/β-catenin signalling through PI3K/Akt [48]. Previous studies have shown that VSMC apoptosis can promote vessel remodeling after carotid ligation [5], and diabetic vein graft remodeling is associated with a simultaneous increase in proliferation and apoptosis of VSMCs [49, 50], although in most cases the underlying mechanisms are not clear.

While our studies implicate cytokines such as IL-6 and GM-CSF derived from apoptotic VSMCs in AICP, apoptotic VSMCs also release chemotactic factors (MCP-1 and M-CSF [5]), and macrophages accumulate after VSMC apoptosis in atherosclerosis [1, 13]; thus, local macrophage production of VSMC mitogens might also promote VSMC AICP. However, we find that intimal or medial AICP does not occur after VSMC apoptosis in atherosclerosis either induced acutely or chronically[1, 13], implying that VSMCs in atherosclerosis are resistant to AICP.

Our study has a number of limitations. First, the conditioned media experiments cannot fully recapitulate the exposure of live VSMCs to apoptotic VSMCs in vivo, as only small molecular weight soluble cytokines are present, excluding the effects of membrane-bound death ligands for example, or proteins in exosomes. Second, we cannot identify a specific cytokine that is responsible for either AICP or AIA; rather we predict that the both processes may be stimulated simultaneously by the concerted effect of multiple secreted cytokines, with the outcome (death or proliferation) being regulated by the precise cytokine combination. Third, the same cytokine relased from apoptotic cells might induce proliferation or apoptosis or protect against apoptosis in adjacent cells, dependent upon its local concentration. Finally, we did not identify AIA after injury in the carotid ligation model using TUNEL, as this does not discriminate between DT-induced apoptosis and AIA.

In summary, we show that VSMC apoptosis induces a number of cytokines including IL-6 and GM-CSF, through pathways that require p38 but not JNK and caspases. VSMC apoptosis can induce both apoptosis or cell proliferation in adjacent live VSMCs, identifying that both VSMC apoptosis-induced apoptosis and apoptosis induced compensatory proliferation may occur in vascular development and disease.

## Electronic supplementary material

Below is the link to the electronic supplementary material.

Supplementary file1 (PDF 3985 kb)

## Data Availability

Data, constructs and mice are available from the corresponding author on request.
