# Supplementary material for: Cytokine regulation of apoptosis-induced apoptosis and apoptosis-induced cell proliferation in vascular smooth muscle cells
Source: Apoptosis. 2020 Jul 5;25(9):648–62. doi: 10.1007/s10495-020-01622-4 (PMC7527356; doi:10.1007/s10495-020-01622-4)
Supplement: Supplementary file 1 — Supplementary file1 (PDF 3985 kb) [file 10495_2020_1622_MOESM1_ESM.pdf]

SUPPLEMENTAL MATERIAL

Cytokine regulation of vascular smooth muscle cell apoptosis-induced apoptosis and cell proliferation

Dimitra Aravani, Kirsty Foote, Nichola Figg, Alison Finigan, Anna Uryga, Murray Clarke, Martin Bennett*,

Division of Cardiovascular Medicine, University of Cambridge
Box 110, ACCI, Addenbrooke's Hospital, Cambridge, CB2 0QQ, UK

Supplemental Figures

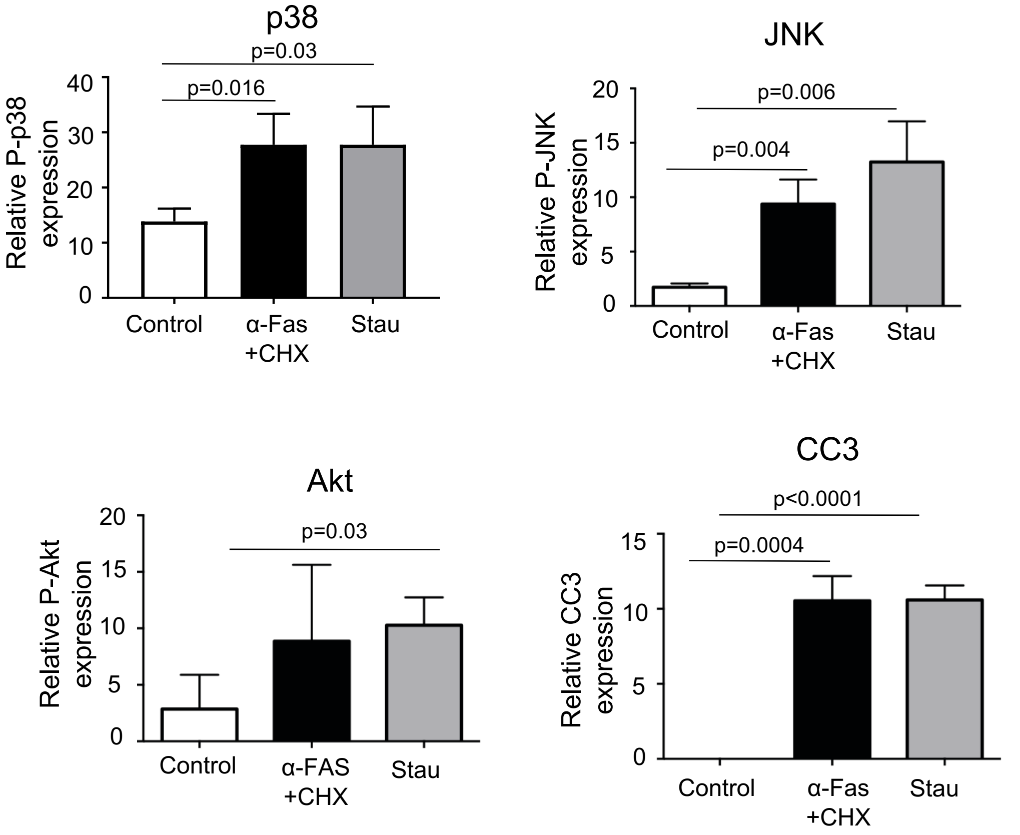

Supplemental Fig. 1
Scanning densitometric analysis of area under the curve over 24h of western blots of relative expression of P-p38/total p38 (normalized to 2h), P-JNK/total JNK, P-Akt/total Akt, or CC3. Data are means ± SEM. n=3.

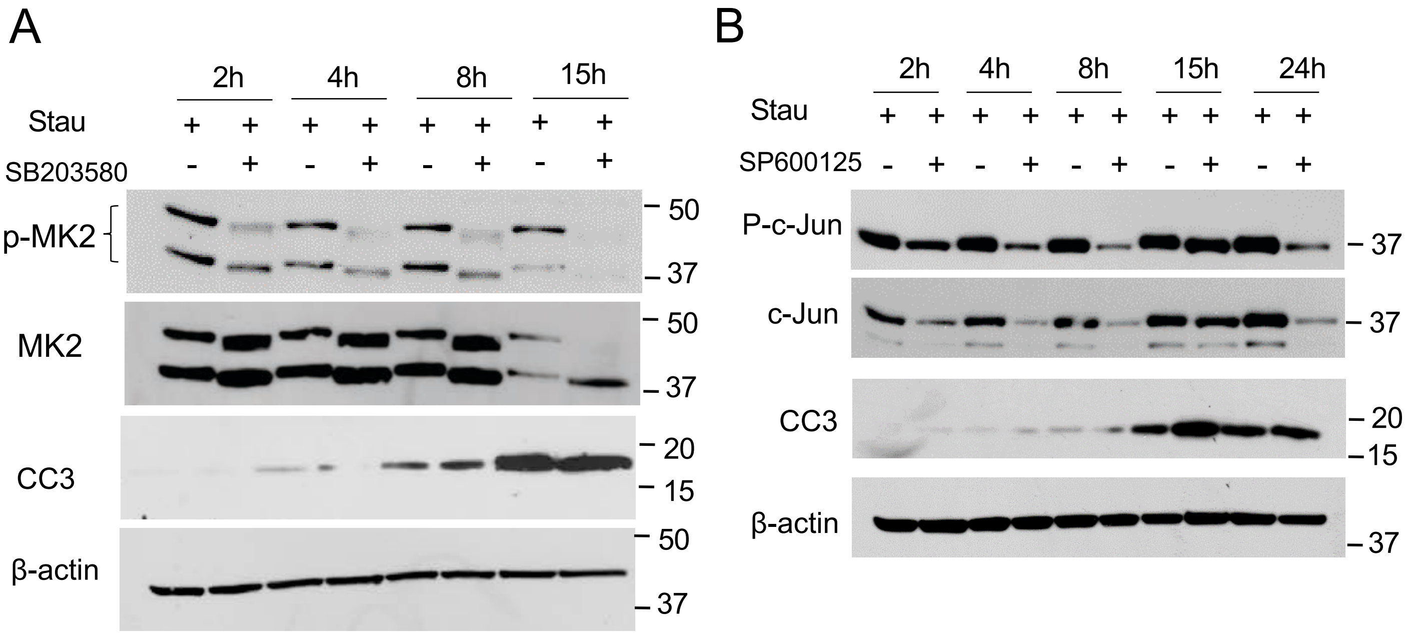

Supplemental Fig. 2
a Western blot of relative expression of p-MK2 and total MK2 in VSMCs treated with Stau for 2-15h ± 10μM p38 inhibitor SB203580. **b** Western blot of relative expression of P-c-Jun and total c-Jun in VSMCs treated with Stau for 2-24h ± 25μM JNK inhibitor SP600125. Data are means ± SD, n=3.

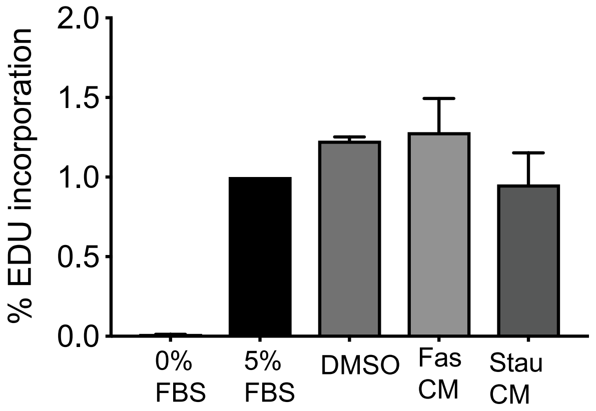

Supplemental Fig. 3
EDU incorporation of growth-arrested VSMCs after treatment with media containing 5% FBS alone or added to conditioned media from VSMCs induced to undergo apoptosis by α-Fas+CHX or Stau or DMSO control. Data are means ± SEM. n=3.

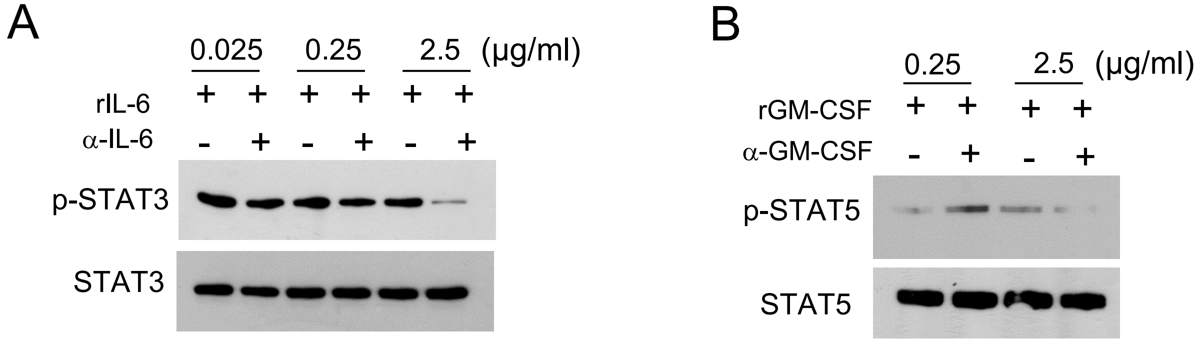

Supplemental Fig. 4
a Western blot for p-STAT3 and total STAT3 after treatment with 50ng/ml recombinant IL-6 ± increasing concentrations of a neutralizing antibody to IL-6. **b** Western blot for p-STAT5 and total STAT5 after treatment with 50ng/ml recombinant GM-CSF ± increasing concentrations of a neutralizing antibody to GM-CSF.

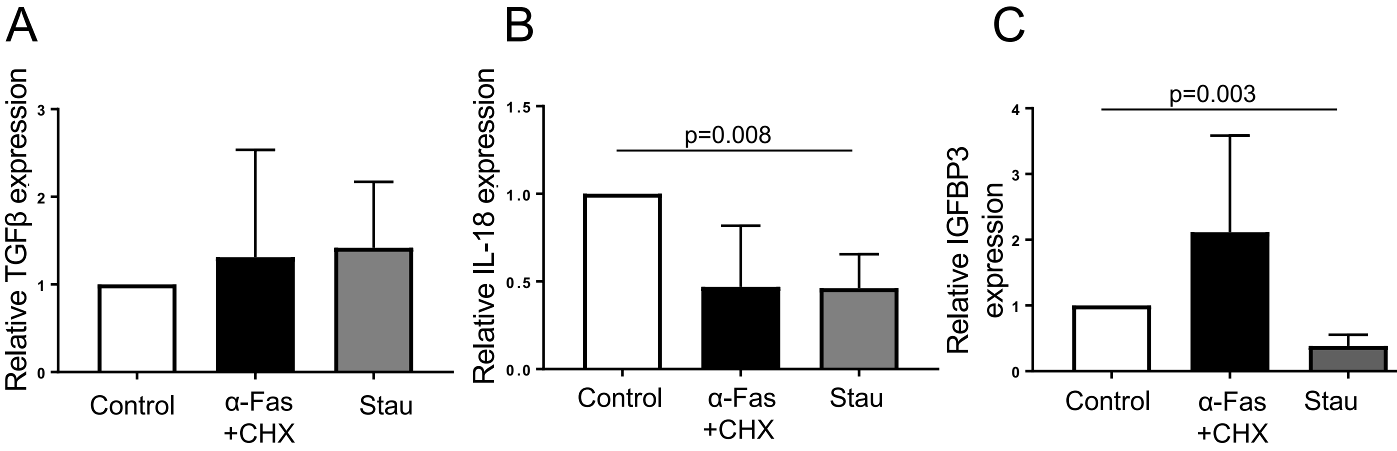

Supplemental Fig. 5
a-c qPCR of TGF-β, IL-1β and IGFBP3 mRNA in Control, α-FAS/CHX and Stau-treated cells following removal of stimuli for 15hours relative to 18S. Data are means ± SD, n=3-5.
